# Prediction of Self-Interacting Proteins from Protein Sequence Information Based on Random Projection Model and Fast Fourier Transform

**DOI:** 10.3390/ijms20040930

**Published:** 2019-02-21

**Authors:** Zhan-Heng Chen, Zhu-Hong You, Li-Ping Li, Yan-Bin Wang, Leon Wong, Hai-Cheng Yi

**Affiliations:** 1 The Xinjiang Technical Institute of Physics and Chemistry, Chinese Academy of Sciences, Urumqi 830011, China; chenzhanheng17@mails.ucas.ac.cn (Z.-H.C.); Lipingli@ms.xjb.ac.cn (L.-P.L.); wangyanbin15@mails.ucas.ac.cn (Y.-B.W.); huangliguang18@mails.ucas.ac.cn (L.W.); yihaicheng17@mails.ucas.ac.cn (H.-C.Y.); 2 University of Chinese Academy of Sciences, Beijing 100049, China

**Keywords:** self-interacting proteins, position-specific scoring matrix, fast Fourier transform, random projection

## Abstract

It is significant for biological cells to predict self-interacting proteins (SIPs) in the field of bioinformatics. SIPs mean that two or more identical proteins can interact with each other by one gene expression. This plays a major role in the evolution of protein‒protein interactions (PPIs) and cellular functions. Owing to the limitation of the experimental identification of self-interacting proteins, it is more and more significant to develop a useful biological tool for the prediction of SIPs from protein sequence information. Therefore, we propose a novel prediction model called RP-FFT that merges the Random Projection (RP) model and Fast Fourier Transform (FFT) for detecting SIPs. First, each protein sequence was transformed into a Position Specific Scoring Matrix (PSSM) using the Position Specific Iterated BLAST (PSI-BLAST). Second, the features of protein sequences were extracted by the FFT method on PSSM. Lastly, we evaluated the performance of RP-FFT and compared the RP classifier with the state-of-the-art support vector machine (SVM) classifier and other existing methods on the *human* and *yeast* datasets; after the five-fold cross-validation, the RP-FFT model can obtain high average accuracies of 96.28% and 91.87% on the *human* and *yeast* datasets, respectively. The experimental results demonstrated that our RP-FFT prediction model is reasonable and robust.

## 1. Introduction

Protein is an important component of all cells. It is an organic macromolecule and the basic material of life. It also is the main undertaker of activity. Without protein, there is no life. Most proteins often work together with a partner or other proteins. They can interact with two or more copies by themselves, which is termed self-interacting proteins (SIPs). However, for most researchers, whether proteins can interact with each other is a difficult thing to determine. SIPs play a key role in the development of protein interaction networks (PINs) [1,2]. The functions of many proteins, which could control the transport of ions and small molecules that pass through cell membranes, depends on their homo-oligomers [3]. Ispolatov et al. discovered that the average quantity of SIPs is more than twice that of other proteins in the PINs [4]. It is crucial for elucidating the functions of SIPs to comprehend whether a protein can self-interact; this also gives us an insight into the adjustment of protein function and can help us achieve a better comprehension of disease mechanisms [5]. Over the past few years, many studies have shown that homo-oligomerization plays an important role in many biological processes, such as signal transduction, gene expression regulation, immune response, and enzyme activation [6,7,8,9]. Therefore, SIPs will be useful for improving steadiness and preventing against cellular stress and the denaturation of proteins via reducing the surface area [10].

So far, there are many ways to study bioinformatics [11,12,13,14,15,16] and genomics [17,18,19,20,21,22], and a number of previous methods for predicting PPIs have been put forward. For example, Pitre et al. [23] raised a new Protein‒Protein Interaction Prediction Engine (PIPE), which could predict PPIs for any target pair of the *yeast S. cerevisiae* proteins from their primary structure and without the need for any additional information or predictions about the proteins. Xia et al. [24] put forward a sequence-based multi-classifier system that applied auto-correlation descriptor to encode a protein interaction pair and selected rotation forest as classifier to deduce PPIs. However, these methods are good for PPI detection [25] but have certain limitations in that they must take the correlation between protein pairs into account for Protein Self-interaction detection—for example, co-expression, co-localization, and co-evolution. Nevertheless, this information is useless for SIPs. Moreover, the datasets for PPI detection are balanced and those of SIPs are unbalanced. Besides, prediction of PPIs datasets has no PPIs between the same partners. For these reasons, the above computational models are not suitable for SIPs detection. Accordingly, it is becoming more and more crucial to exploit an effective calculation method to predict SIPs.

In our study, a random projection (RP) method for SIPs prediction from protein sequence information with Fast Fourier Transform (FFT) was proposed. Furthermore, the main idea of our proposed method includes four aspects: (1) the protein sequence information could be described as a Position-Specific Scoring Matrix (PSSM); (2) using the fast Fourier transform (FFT) method to extract eigenvectors from protein sequences on a PSSM; (3) using the Principal Component Analysis (PCA) approach to convert the high-dimensional data into useful information after FFT and the noise is removed, so the pattern in the data is found; (4) the RP algorithm is employed to build a training set where the classifier will be trained. Take it in detail as follows: first, the PSSM from each protein sequence is likely to result in a eigenvector whose dimension is 400 by applying the FFT method for extracting important information; then, reduce the dimension of the FFT vector to 300 for improving the performance of prediction by employing the PCA dimensionality reduction method; eventually, perform classification on *yeast* and *human* datasets by applying the RP classifier. The results demonstrate that this method outperforms the SVM-based approach and six other existing technologies. This indicates that the proposed model is suitable and performs well for predicting SIPs.

## 2. Results and Discussion

### 2.1. Performance Evaluation

In this study, to estimate the stability and availability of our prediction model, we used five measurements that were commonly used in binary classification tasks, including accuracy (Acc.), sensitivity (Sen.), specificity (Spe.), Matthews correlation coefficient (MCC) [26,27,28,29,30,31,32], and Balanced Accuracy (B_Acc.) [33], respectively. They could be defined as follows:

(1) $$Acc=\frac{TP+TN}{TP+TN+FP+FN}$$

(2) $$Sen=\frac{TP}{TP+FN}$$

(3) $$Spe=\frac{TN}{TN+FP}$$

(4) $$MCC=\frac{\left(TP\cdot TN)-(FP\cdot FN\right)}{\sqrt{\left(TP+FN)(TN+FP)(TP+FP)(TN+FN\right)}}$$

(5) $$B\_\mathrm{Acc}=\frac{Sen+Spe}{2}=\frac{2TP\cdot TN+TP\cdot FP+TN\cdot FN}{2(TP+FN)(TN+FP)},$$

where *TP* represents the count of true positives, that is to say the number of real interacting pairs predicted correctly. *FP* is the quantity of false positives, defined as the volume of real non-interacting pairs mis-predicted. *TN* stands for the count of true negatives, which is the quantity of real non-interacting pairs correctly predicted. *FN* means the quantity of false negatives; in other words, it represents the true sample error predicted to be false samples. On the basis of these parameters, a Receiver Operating Curve (ROC) was plotted to assess the performance of the random projection approach. Then we can compute the area under the curve (AUC) to estimate the quality of the classifier.

### 2.2. Performance of the Proposed Method

In order to evaluate the performance of the presented model and avoid the overfitting problem, we applied the RP-FFT model to the *human* dataset. In statistical prediction, three cross-validation (CV) methods, such as an independent dataset test, a sub-sampling (or k-fold CV) test, and a leave-one-out CV (LOOCV) test, are frequently used to calculate the expected success rate of a developed predictor [34,35,36,37,38]. Among the three methods, however, the LOOCV test is deemed the least arbitrary and most objective, as demonstrated by Equations (28)–(32) of [39], and hence it has been widely recognized and increasingly adopted by investigators to examine the quality of various predictors [38,40,41]. However, it seems time- and resource-consuming. Thus, we used 5-fold CV to examine the proposed models. In 5-fold CV, the benchmarking dataset was randomly partitioned into 10 subsets. One subset is used as a test set and the remaining nine subsets are used as the training sets. This procedure is repeated five times, where each subset is used once as a test set. The performance of the five corresponding results is averaged to give the performance of the classifier. To assess the feasibility and stability of our prediction method, we also estimated the prediction performance of RP-FFT model on the *yeast* dataset.

To ensure the fairness of the experiment, we optimized a number of parameters for the RP-FFT prediction model. In this paper, we set up the same parameters for *human* and *yeast* datasets. Thus, we classify the training and test sets for *B*1 = 10 independent projections, each one carefully chosen from a block of size *B*2 = 30, and then chose the K-Nearest Neighbor (KNN) as the base classifier and the leave-one-out test error estimate, where *k* = *seq* (1, 40, by = 3).

Our model can not only deal with balanced data, but can also solve the imbalanced data problem to some extent. At first, we employed the undersampling technique, as mentioned in [18], to solve the imbalanced dataset problem. The *human* dataset included 1441 SIPs as positives and 1441 non-SIPs as negatives. Using the same strategy, the *yeast* dataset contained 710 positive samples and 710 negative samples. The experimental results can be seen in Table 1 and Table 2. 

In addition, the initial imbalanced data collected from DIP, BioGRID, IntAct, InnateDB, and MatrixDB also used to compare our proposed method with previous work. If we use the undersampling technique to reconstruct the dataset, the size of the initial imbalanced data will be substantially reduced. As shown in Table 2 and Table 3, we performed our proposed model on the initial imbalanced data in the experiment.

The experimental results of the RP-FFT prediction model on the *human* and *yeast* datasets are listed in Table 3 and Table 4. Table 3 lists the data obtained that the model put forward obtained for average Accuracy (Acc.), Sensitivity (Sen.), Specificity (Spe.), Matthews correlation coefficient (MCC), and Balance accuracy (B_Acc.): 96.28%, 81.48%, 97.62%, 76.46%, and 89.55% for the *human* dataset and the standard deviations of them 0.22%, 2.43%, 0.35% 1.29%, and 1.08%, respectively. In the same way, we also got good results in Table 4 for average Acc., Sen., Spe., MCC and B_Acc.: 91.87%, 48.81%, 97.42%, 54.62% and 73.12%, and the standard deviations of them are 0.82%, 4.50%, 0.45%, 4.25%, and 2.30% for the *yeast* dataset, respectively.

From the above data, it is obvious that the proposed method could achieve good outcomes for SIPs predictions due to the suitable feature extraction and classifier. It can be summarized that the main improvement of our characteristic extraction technique contains the following factors: (1) The PSSM gives the score for finding a special matching amino acid in a target protein sequence. It is a good tool that can not only represent the protein sequence information but also saves enough prior information. Therefore, a PSSM contains all the major information of one protein sequence for detecting SIPs. (2) We extracted the features from the protein sequence by using the Fast Fourier Transform (FFT) method, which can further increase the performance of the RP-FFT model. (3) In case of ensuring the integrity information of FFT feature vector, we used Principal Component Analysis (PCA) to decrease the dimension of data and influence of noise, and thus the pattern in the data is found. Experimental results revealed that the eigenvector extracted from applying FFT on PSSM is quite suitable for SIP detection.

### 2.3. Comparison with Other Feature Extraction Methods

In this section, in order to illustrate the use of the FFT feature extraction method, we compared the FFT method with SVD (Singular Value Decomposition), DCT (Discrete Cosine Transform), and COV (Covariance) [42,43] on the Random Projection classifier. The results of RP classifier based on different feature extraction methods with 5-fold cross-validation on the *yeast* dataset are shown in Table 5. On the whole, it can be seen that the FFT feature extraction method works better than other methods for the *yeast* dataset.

### 2.4. Comparison with the SVM-Based Method

Though the RP-FFT model achieved better performance for predicting SIPs, we still need to further assess its use with our presented method. The veracity and stability of prediction of the RP classifier were compared with the state-of-the-art SVM method via the same characteristic extraction approach based on the *yeast* and *human* datasets, respectively. We applied the LIBSVM packet tool [44] to run the classification. Before the experiment, there are several parameters of SVM classifier should be optimized. In this paper, we chose a radial basis function (RBF) as the kernel function, and then used grid search to optimize the parameters of RBF, whose parameters were set to *c* = 0.03 and *g* = 1200.

As shown in Table 6 and Table 7, we employed 5-fold cross-validation to train and compare the models of RP and SVM on the *yeast* and *human* datasets, respectively. The average Acc., the average Sen., the average Spe., the average MCC and B_Acc. of SVM classifier are 93.68%, 23.80%, 100.00%, 47.13%, and 61.90% on the *human* dataset in Table 6, respectively. Nevertheless, the RP classifier obtained 96.28% average Acc., 81.48% average Sen., 97.62% average Spe., 76.46% average MCC, and 89.55% average B_Acc. On the *human* dataset. Similarity, the average Accuracy, the average Sen., the average Spe., the average MCC and B_Acc. of SVM classifier are 90.63%, 17.79%, 100.00%, 39.95%, and 58.90% on the *yeast* dataset in Table 7. Nevertheless, the RP classifier received 91.87% average Acc., 48.81% average Sen., 97.42% average Spe., 54.62% average MCC and 73.12% average B_Acc. On the *human* dataset. In a word, it is obvious that the overall prediction result of RP classifier is much better than that of the SVM method.

**Figure 1:**
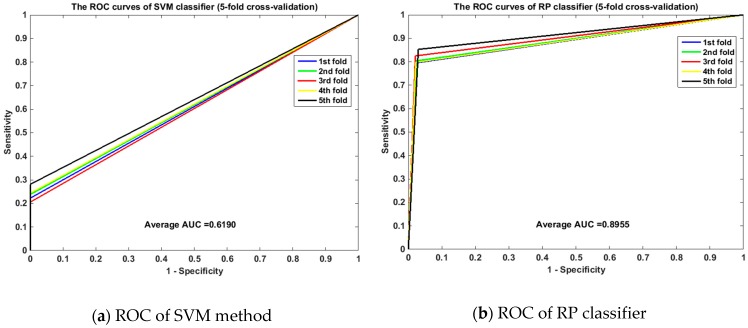
Comparison of ROC curves between RP and SVM on *human* (5-fold cross validation). (**a**) is the ROC curve of SVM method on *human* dataset by 5-fold cross validation. (**b**) is the ROC curve of RP classifier on *human* dataset by 5-fold cross validation.

**Figure 2:**
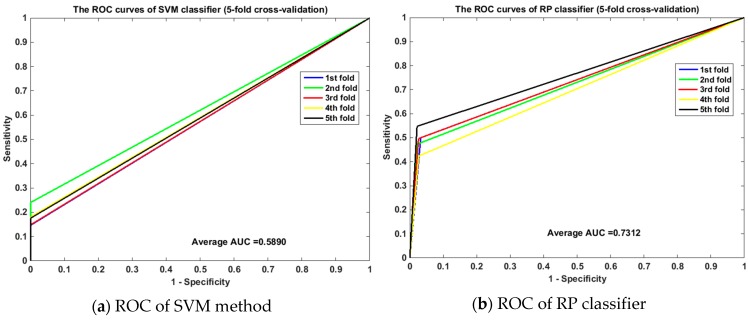
Comparison of ROC curves between RP and SVM on *yeast* (5-fold cross validation). (**a**) is the ROC curve of SVM method on *yeast* dataset by 5-fold cross validation. (**b**) is the ROC curve of RP classifier on *yeast* dataset by 5-fold cross validation.

Meanwhile, the ROC curves between RP and SVM on the *human* and *yeast* datasets are displayed in Figure 1 and Figure 2. From Figure 1, it is clear that the average area under the curve (AUC) of SVM classifier is 0.6190 and that of the RP classifier is 0.8955. From Figure 2, we can see that the average AUC of SVM classifier is 0.5890 and that of the RP classifier is 0.7312. It is obvious that the average AUC of RP method is also larger than the AUC of the SVM method. So Random Projection is an accurate and robust method for SIP detection.

### 2.5. Comparison with Other Existing Methods

In our study, we compared the presented model, termed RP-FFT, with other existing models on the *yeast* and *human* datasets to further prove that it can achieve good results. These comparison results of RP-FFT models and other models on the *yeast* and *human* datasets are shown in Table 8 and Table 9. From Table 8, it is obvious that the RP-FFT model obtained a higher average accuracy than other existing models on *yeast* dataset. It is also clear that the other six methods got lower specificity and sensitivity than our proposed model for the same dataset. Accordingly, as is apparent from Table 9, the overall outcomes of our prediction model are also significantly better than the other six models on the *human* dataset. To sum up, the experimental results of the proposed model called RP-FFT prove its accuracy for predicting SIPs compared with the six approaches. This explains why our prediction model is superior to the other six methods, because it employs a good method of feature extraction and a suitable classifier. It can be further illustrated that our RP-FFT model is suitable for predicting SIPs.

## 3. Materials and Methodology

### 3.1. Datasets

The datasets derived from the UniProt database [49] include 20,199 curated *human* protein sequences. The PPIs data could be collected from a variety of sources, including DIP [50], BioGRID [51], IntAct [52], InnateDB [53], and MatrixDB [54]. In this experiment, we mainly built the PPIs dataset, which obtains two identical interacting proteins and whose style of interaction was described as ‘direct interaction’ in correlative databases. On this foundation, 2994 *human* SIPs could be obtained.

We built the datasets to estimate the performance of our prediction method, which has three steps [48]: (1) protein sequences with a length less than 50 or more than 5000 residues from the *human* proteome were removed; (2) to build the *human* positive dataset, we picked out the SIPs data with high quality, which should meet one of the following requirements: (a) the self-interactions were discovered by at least one small-scale experiment or two types of large-scale experiments; (b) we annotated the protein as a homo-oligomer (comprising homodimer and homotrimer) in UniProt; (c) it has been reported by at least two publications for self-interactions; (3) for the *human* negative dataset, we eliminated SIPs from all the *human* proteome (containing proteins labeled as ‘direct interaction’ and much wider ‘physical association’) and the prediction of SIPs in the UniProt database. Eventually, the *human* dataset contained 1441 SIPs as a positive dataset and 15,938 non-SIPs as a negative dataset [48].

In addition, the *yeast* dataset was also built to further illustrate the cross-species performance of the RP-FFT model, which included 710 SIPs samples and 5511 non-SIPs samples [48] via the same strategy mentioned above.

### 3.2. Position-Specific Scoring Matrix

We discovered distantly correlative proteins by applying the Position-Specific Scoring Matrix (PSSM) [55,56,57], which is a helpful tool. Therefore, a PSSM can be transformed from each protein sequence information by applying the Position-Specific Iterated BLAST (PSI-BLAST) [58]. Then, each protein sequence could be transformed into an *N* × 20 PSSM matrix as follows:

(6) $$M=\{M_{\alpha \beta },\;\alpha =1,\cdots ,N,\;\beta =1,\cdots ,20\},$$

where *N* indicates the size of a protein sequence, and each protein gene was constructed by 20 types of amino acids. For the query protein sequence, a PSSM could arrange the value *M_αβ_* that represents the *β*-th amino acid at the position of *α*. Thus, *M_αβ_* could be described as:

(7) $$M_{\alpha \beta }=\sum_{k=1}^{20}p(\alpha ,k)\times q(\beta ,k),$$

where *p*(*α*, *k*) means the occurrence frequency score of the *k*-th amino acid in the position of *α* with the probe, and *q*(*β*, *k*) represents the value of Dayhoff’s mutation matrix between the *β*-th and *k*-th amino acids. Accordingly, a high value is a strongly conservative position; otherwise, it means a weakly conservative position.

In conclusion, PSSM could be a helpful tool for predicting self-interacting proteins. Each PSSM from the protein sequence was generated by employing PSI-BLAST for SIPs detection. For the sake of getting a high degree and a wide range of homologous information, we chose three iterations and assigned the e-value of PSI-BLAST to be 0.001 in this process. Consequently, the PSSM of each protein sequence could be expressed as a matrix consisting of *M* × 20 elements, where row *M* of the matrix means the quantity of residues of each protein, and column 20 of the PSSM indicates the 20 different kinds of amino acids.

### 3.3. Fast Fourier Transform

Fast Fourier Transform (FFT) [59] was first applied in digital signal processing in a number of diverse areas. Afterwards it was used for image processing for a given curve *C* whose shape was a closed scope. At a certain time *t*, there is a data sequence *F*(*t*), 0 ≤ *t* < *T*. Since *F*(*t*) is a periodic function, *F*(*t*) = *F*(*t* + *nT*). In this study, we used it to extract the eigen values. Hence, we expand *F*(*t*) into a Fourier series as much as possible; it can be described as follows:

(8) $$F(t)=\sum_{-\infty }^{\infty }\omega_{n}e^{\left(2\alpha \pi nt/T\right)},$$

where *ω_n_* is the Fourier coefficients of *F*(*t*).

(9) $$\omega_{n}=\frac{1}{T}\int_{0}^{T}F(t)e^{\left(-2\alpha \pi nt/T\right)}dt,\;n\in \text{ℤ}$$

The discrete Fourier transform is given by

(10) $$\omega_{n}=\frac{1}{N}\sum_{t=0}^{N-1}F(t)e^{\left(-2\alpha \pi nt/N\right)},\;n=0,1,\cdots ,N-1,$$

where $\alpha =\sqrt{-1}$, $N=2^{n}$, $n=1,2,\cdots ,n_{\max}$. *F*(*t*) is commonly named the shape signature, which represents the shape boundary of any one-dimensional function. Fourier transform could only capture the architectural characteristics of a shape, which is important to stem FFT from a perceptually meaningful shape signature. FFT stemmed from the centroid distance function is superior to FFT stemmed from other shape signatures. From the centroid (*x_c_*, *y_c_*) of the shape, the centroid distance function *r*(*t*) could be defined by the distance of the boundary points:

(11) $$r(t)={\left({\left[x(t)-x_{c}\right]}^{2}+{\left[y(t)-y_{c}\right]}^{2}\right)}^{1/2},$$

where $x_{c}=\frac{1}{N}\sum_{t=0}^{N-1}x(t)$, $y_{c}=\frac{1}{N}\sum_{t=0}^{N-1}y(t)$ and *N* is the quantity of boundary points.

It is a matter of great significance to extract informative characteristics based on machine learning approaches. In our study, for the sake of each protein sequence being composed of amounts of amino acids, the eigenvector cannot be directly obtained from a PSSM by PSI-BLAST, which will lead to diverse length of eigenvectors. For solving the question, we multiply the transpose of PSSM by PSSM to obtain a 20 × 20 matrix, and the feature extraction method of fast Fourier transform is employed to generate characteristic vectors from the PSSM profile. In the end, each protein sequence could be calculated to a 400-dimensional vector after FFT. In this study, eventually, each protein sequence from the *yeast* and *human* datasets was transformed into a 400-dimensional vector by employing the fast Fourier transform method.

In our study, for the sake of obtaining the main important data and advancing the prediction accuracy, we used the Principal Component Analysis (PCA) approach to reduce the size of the *yeast* and *human* databases from 400 to 300. Furthermore, reducing the dimensionality of the datasets could remove the complexity of the classifier and improve the generalization error.

### 3.4. Support Vector Machine

Support vector machine (SVM) was first proposed by Cortes and Vapnik et al. [60] in 1995. SVM inherently do binary classification. SVM is a statistical learning theory method, which is mainly used in the field of pattern recognition. The purpose of SVM is to find the hyperplane that maximizes the distance margin between the two classes. Hence, we can transform it into a convex quadratic programming problem. This idea can be expressed formally as follows:

(12) $$\begin{array}{cc} \underset{w,b,\xi }{\min} & \frac{w^{T}w}{2}+C\sum_{i=1}^{l}\xi_{i}\\ \text{subject to} & y_{i}(w^{T}\emptyset (x_{i})+b)\geq 1-\xi_{i},\\ & \xi_{i}\geq 0 \end{array}$$

where (*x_i_*, *y_i_*) is a training set of instance-label pairs, *i* = 1, ..., *l*. *x_i_* ϵ *R^n^* are mapped into a higher dimensional space by the function Ø. *y* ϵ {1, −1}*^l^*. Furthermore, the kernel function can be described as *K*(*x_i_*, *x_j_*) ≡ Ø(*x_i_*)*^T^*Ø(*x_j_*). It has four basic kernels that can be found in [61]:

(1) Linear: *K*(*x_i_*, *x_j_*) = $x_{i}^{T}$*x_j_*.
(2) Polynomial: *K*(*x_i_*, *x_j_*) = (*γ*$x_{i}^{T}$*x_j_* + *r*)*^d^*, *γ* > 0.
(3) Radial basis function (RBF): *K*(*x_i_*, *x_j_*) = exp(−*γ*||*x_i_* − *x_j_*||^2^), *γ* > 0.
(4) Sigmoid: *K*(*x_i_*, *x_j_*) = tan *h*(*γ*$x_{i}^{T}$*x_j_* + *r*).

Here, *γ*, *r*, and *d* are kernel parameters. In our experiment, we chose RBF as the kernel function.

### 3.5. Random Projection Classifier

In mathematics and statistics, random projection (RP) is a technique for dimensionality reduction of some points that exist in Euclidean space. The meaning of the RP method is that projecting *N* points in *N* dimensional space can almost always onto a space of dimension *ClogN* with control on the ratio of distances and the error [62]. This method has been successfully applied for the reestablishment of frequency-sparse signals [63,64], facial recognition [65,66,67], protein mapping [68], and textual and visual information retrieval [69].

Next, we formally describe the random projection technique in detail. First, let

(13) $$\Gamma ={\left\{A_{i}\right\}}_{i=1}^{N},\;A_{i}\in R^{n}$$

be the primitive high-dimensional space dataset, where *n* is the quantity of high dimension and *N* is the count of the dataset. The goal of descending dimension is embedding the eigenvectors into a lower dimensional space *R^q^* from a high-dimensional *R^n^* where *q* << *n*. The output of data is represented as follows:

(14) $$\tilde{\Gamma }={\left\{\tilde{A_{i}}\right\}}_{i=1}^{N},\;\tilde{A_{i}}\in R^{q},$$

where *q* approaches the inherent dimensionality of Γ. Thus, the vectors of Γ were regarded as embedding vectors.

If we want to reduce the dimension of Γ via the random projection method, a random vector set *γ* = ${\left\{r_{i}\right\}}_{i=1}^{k}$ must first be constructed, where *r_i_* ∈ *R^q^*. The random basis can be obtained by two common choices, as follows [62]:

(1) The vectors ${\left\{r_{i}\right\}}_{i=1}^{k}$ are normally distributed over the *q* dimensional unit sphere.
(2) The components of the vectors ${\left\{r_{i}\right\}}_{i=1}^{k}$ are selected Bernoulli +1/−1 distribution and the vectors are standardized so that ||*r_i_*||*_l_*_2 = 1_ for *i* = 1, …, *n*.

The columns of *q* × *n* matrix *R* consist of the vectors in *γ*. The embedding result *Ã_i_* of *A_i_* can be got by

(15) $$\tilde{A_{i}}=R\cdot A_{i}$$

In our proposed method, random projection is employed to build a training set where the classifier would be trained. We enrich the component of the integration method by using random projection.

Next, the dimension of the objective space was set to one part around the space where the training members reside. We built a size of *n* × *N* matrix *G* whose columns are made up the column eigenvectors in Γ. The training set Γ is given in Equation (7).

(16) $$G=\left(A_{1}|A_{2}|\ldots |A_{N}\right)$$

Then, we construct *k* random matrices ${\left\{R_{i}\right\}}_{i=1}^{k}$ whose magnitude is *q* × *n*, *q* and *n* are mentioned in the above paragraph, and *k* is the quantity of integration classifiers. Here, the columns of matrices are normalized so the *l*_2_ norm is 1.

Then, using our method, we constructed training sets ${\left\{T_{i}\right\}}_{i=1}^{k}$ by projecting *G* onto ${\left\{R_{i}\right\}}_{i=1}^{k}$ which is the *k* random matrix. It can be represented as follows:

(17) $$T_{i}=R_{i}\cdot G,\;i=1,\ldots ,k.$$

The training sets are imported into an inducer and the export results are a set of classifiers ${\left\{\ell_{i}\right\}}_{i=1}^{k}$. How do we classify a new dataset *I* through classifier *ℓ_i_*? First, we embed *I* into the dimensionality reduction space *R^q^*. Then, it can be owned via embedding *u* in the random matrix *R_i_* as follows:

(18) $$\tilde{I}=R_{i}\cdot I,$$

where *Ĩ* is the inlaying of *u*, the classification of *Ĩ* can be garnered from the classification of *I* by *ℓ_i_*. In this ensemble method, the random projection classifier apply a data-driven voting threshold that is employed on the classification results of the whole classifier ${\left\{\ell_{i}\right\}}_{i=1}^{k}$ for the *Ĩ* to decide produce the ultimate classification result of *Ĩ*.

In this experiment, the random projections were segmented into non-overlapping parts, where *B*1 = 10 and each one was carefully chosen from a certain part of size *B*2 = 30 that achieved the smallest estimate of the test error. We chose the k-Nearest Neighbor (KNN) as the base classifier and the leave-one-out test error estimate, where *k* = *seq* (1, 40, by = 3). The prior probability of interaction pairs in the training sample dataset was taken as the voting parameter. Our classifier integrates the results of taking advantage of the base classifier on the chosen projection, with the data-driven voting threshold confirming the ultimate mission.

## 4. Conclusions

In our study, we developed a new prediction model based on protein sequence information to detect SIPs. This model was created by combining Position-Specific Scoring Matrix with Fast Fourier Transform and Random Projection classifier, which was termed RP-FFT. The main point of the experiment is that the datasets used by the classifier are unbalanced. The main improvements of the presented model are: (1) making use of a reasonable feature extraction method that could capture the main information of the data to improve the performance efficiency. (2) The RP classifier is strongly suitable for SIPs prediction. To summarize, the experimental results achieved by the presented method on the *yeast* and *human* datasets indicated that our prediction performance is obviously better than that of the SVM-based method and six other existing models. In the future, there will be more and more characteristic extraction techniques and machine learning or deep learning methods attempted for detecting SIPs.

## Figures and Tables

**Table 1:** The results of the RP-FFT method with 5-fold cross-validation on the *human* dataset.

Testing Set	Acc. (%)	Sen. (%)	Spe. (%)	MCC (%)
1	94.44	88.28	100.00	89.36
2	92.53	85.37	100.00	86.07
3	92.19	85.48	100.00	85.51
4	93.75	86.76	100.00	88.08
5	94.81	89.73	100.00	90.12
Average	93.54 ± 1.15	87.12 ± 1.87	100.00 ± 0.00	87.83 ± 2.01

**Table 2:** The results of the RP-FFT method with 5-fold cross-validation on the *yeast* dataset.

Testing Set	Acc. (%)	Sen. (%)	Spe. (%)	MCC (%)
1	80.99	97.12	65.52	65.71
2	83.45	92.14	75.00	68.03
3	82.04	97.89	66.20	67.57
4	84.86	95.14	74.29	71.13
5	83.45	92.41	74.10	67.83
Average	82.96 ± 1.48	94.94 ± 2.63	71.02 ± 4.73	68.05 ± 1.95

**Table 3:** The results of the RP-FFT method with 5-fold cross-validation on the *human* dataset.

Testing Set	Acc. (%)	Sen. (%)	Spe. (%)	MCC (%)	B_Acc. (%)
1	96.23	79.51	97.74	75.72	88.63
2	96.20	80.34	97.65	75.89	89.00
3	96.58	82.49	97.89	78.61	90.19
4	96.40	79.78	97.79	75.40	88.79
5	96.00	85.28	97.01	76.68	91.15
Average	96.28 ± 0.22	81.48 ± 2.43	97.62 ± 0.35	76.46 ± 1.29	89.55 ± 1.08

**Table 4:** The results of the RP-FFT method with 5-fold cross-validation on the *yeast* dataset.

Testing Set	Acc. (%)	Sen. (%)	Spe. (%)	MCC (%)	B_Acc. (%)
1	91.32	50.00	96.73	53.09	73.37
2	91.72	47.33	97.81	55.35	72.57
3	92.20	49.63	97.39	54.80	73.51
4	91.00	42.36	97.36	49.06	69.86
5	93.09	54.74	97.83	60.82	76.29
Average	91.87 ± 0.82	48.81 ± 4.50	97.42 ± 0.45	54.62 ± 4.25	73.12 ± 2.30

**Table 5:** The results of RP classifier based on different feature extraction methods on the *yeast* dataset.

Feature Extraction Methods	Acc. (%)	Sen. (%)	Spe. (%)	MCC (%)	B_Acc. (%)
SVD	88.73 ± 0.75	10.25 ± 2.93	98.86 ± 0.43	19.76 ± 2.96	54.55 ± 1.31
DCT	90.35 ± 0.84	20.38 ± 2.62	99.36 ± 0.32	37.57 ± 1.74	59.87 ± 1.18
COV	91.93 ± 0.81	42.43 ± 4.82	98.31 ± 0.25	53.10 ± 4.91	70.37 ± 2.49
FFT	91.87 ± 0.82	48.81 ± 4.50	97.42 ± 0.45	54.62 ± 4.25	73.12 ± 2.30

**Table 6:** Performance comparison of RP and SVM on the *human* dataset.

Model	Testing Set	Acc. (%)	Sen. (%)	Spe. (%)	MCC (%)	B_Acc. (%)
RP + FFT	1	96.23	79.51	97.74	75.72	88.63
	2	96.20	80.34	97.65	75.89	89.00
	3	96.58	82.49	97.89	78.61	90.19
	4	96.40	79.78	97.79	75.40	88.79
	5	96.00	85.28	97.01	76.68	91.15
	Average	96.28 ± 0.22	81.48 ± 2.43	97.62 ± 0.35	76.46 ± 1.29	89.55 ± 1.08
SVM + FFT	1	93.55	22.22	100.00	45.57	61.11
	2	93.64	23.79	100.00	47.17	61.90
	3	93.21	20.54	100.00	43.73	60.27
	4	94.19	24.34	100.00	47.86	62.17
	5	93.82	28.09	100.00	51.30	64.05
	Average	93.68 ± 0.36	23.80 ± 2.82	100.00 ± 0.00	47.13 ± 2.82	61.90 ± 1.41

**Table 7:** Performance comparison of RP and SVM on the *yeast* dataset.

Model	Testing Set	Acc. (%)	Sen. (%)	Spe. (%)	MCC (%)	B_Acc. (%)
RP+FFT	1	91.32	50.00	96.73	53.09	73.37
	2	91.72	47.33	97.81	55.35	72.57
	3	92.20	49.63	97.39	54.80	73.51
	4	91.00	42.36	97.36	49.06	69.86
	5	93.09	54.74	97.83	60.82	76.29
	Average	91.87 ± 0.82	48.81 ± 4.50	97.42 ± 0.45	54.62 ± 4.25	73.12 ± 2.30
SVM+FFT	1	90.11	14.58	100.00	36.22	57.29
	2	90.84	24.00	100.00	46.62	62.00
	3	90.76	14.81	100.00	36.64	57.41
	4	90.51	18.06	100.00	40.38	59.03
	5	90.92	17.52	100.00	39.87	58.76
	Average	90.63 ± 0.33	17.79 ± 3.80	100.00 ± 0.00	39.95 ± 4.17	58.90 ± 1.90

**Table 8:** Comparison of RP-FFT with the other existing models on the *yeast* dataset.

Model	Acc. (%)	Spe. (%)	Sen. (%)	MCC (%)	B_Acc. (%)
SLIPPER [2]	71.90	72.18	69.72	28.42	70.95
DXECPPI [45]	87.46	94.93	29.44	28.25	62.19
PPIevo [46]	66.28	87.46	60.14	18.01	73.80
LocFuse [47]	66.66	68.10	55.49	15.77	61.80
CRS [48]	72.69	74.37	59.58	23.68	66.98
SPAR [48]	76.96	80.02	53.24	24.84	66.63
Proposed method	91.87	97.42	48.81	54.62	73.12

**Table 9:** Comparison of RP-FFT with the other existing models on the *human* dataset.

Model	Acc. (%)	Spe. (%)	Sen. (%)	MCC (%)	B_Acc. (%)
SLIPPER [2]	91.10	95.06	47.26	41.97	71.16
DXECPPI [45]	30.90	25.83	87.08	8.25	56.46
PPIevo [46]	78.04	25.82	87.83	20.82	56.83
LocFuse [47]	80.66	80.50	50.83	20.26	65.67
CRS [48]	91.54	96.72	34.17	36.33	65.45
SPAR [48]	92.09	97.40	33.33	38.36	65.37
Proposed method	96.28	97.62	81.48	76.46	89.55
